# LGBTQ Health Research: Theory, Methods, Practice

**DOI:** 10.5195/jmla.2021.1238

**Published:** 2021-07-01

**Authors:** Andy Hickner

**Affiliations:** 1 alh4014@med.cornell.edu, Weill Cornell Medicine, New York, NY

*LGBTQ Health Research: Theory, Methods, Practice* is a primer on public health and behavioral research methods involving LGBTQ (lesbian, gay, bisexual, transgender, queer or questioning) populations. Medical research is mostly beyond the book's scope, although clinical and pharmacy researchers may find chapters 4, 5, and 7 relevant; these chapters address population definitions, sampling considerations, and measurement. It appears that none of the authors are based outside of the US, although there are a couple chapters that discuss global health and considerations for research in other countries.

The book is divided into three sections. The first, “Introduction to LGBTQ Health Research,” includes chapters on the human rights context and global health, as well as the chapter “A Love Note to Future Generations of LGBTQ Health Researchers,” which offers encouragement to current and future researchers and stresses the importance of mentorship.

The second section, “Descriptive Research Methods,” is in this reviewer's opinion the most broadly useful to researchers in other fields of health research. It includes chapters on the challenges of defining and measuring the complex concepts of sexual orientation and of gender identity, practical approaches to surmounting the challenges of sampling LGBTQ populations, how to incorporate theoretical frameworks into study design, and approaches involving social networks and couples in HIV prevention and care.

The third section, “Intervention Design and Research,” focuses on designing and carrying out public health interventions. Rich in case studies, it addresses topics including how to engage and target populations, involving members of the target population in the design and implementation of programs, and the use of systematic program-planning processes to ensure programs are responsive to the communities they are designed to serve. As with sampling, effectiveness and implementation research in LGBTQ populations is particularly challenging; the authors outline an agenda for addressing these challenges.

As the book notes, most of the published research in LGBTQ populations has focused on HIV/AIDS among gay men in high-income countries, with white, urban, cisgender gay men overrepresented in study populations. One of the book's strengths is its consistent emphasis on intersectionality and the necessity of tailoring approaches in order to account for differences across ethnic, cultural, socioeconomic, and geographic populations. The authors repeatedly remind readers that in most of the world, LGBTQ people must conceal their behavior and identity due to criminal laws and/or social stigma, and that even in the US a significant portion of LGBTQ people face stigma and discrimination, which pose unique obstacles for those seeking to study these groups. Throughout the book, the authors stress the heterogeneity of LGBTQ populations and how methods designed to study sexual behavior in mostly urban white gay men are often not transferrable to lesbian, transgender, bisexual, rural, and nonwhite populations.

Although the book cites examples of studies on other topics such as smoking and alcohol use, the largest number of examples involve HIV/AIDS and safe sex, presumably since, as the authors note, the overwhelming majority of published literature in LGBTQ populations is on this topic. Given the number of examples pertaining to sexual health in the book, it is somewhat surprising that research on pre-exposure prophylaxis (PrEP), which has in some ways transformed the sexual behavior of gay men and trans people over the past decade, is only briefly mentioned.

A search of WorldCat shows this book is one of a dozen or so handbooks on research involving sexual and gender minorities in other fields such as law and psychology, mostly in the American context. While the main audience for this book is likely to be health behavior researchers and graduate students in the United States, some chapters will be useful to researchers in other parts of the world for which such a handbook does not yet exist. As the chapters on human rights and global health make apparent, the opportunity is ripe for future researchers to address the relative scarcity of methodological guidance regarding LGBTQ populations outside high-income Western countries.

